# Characterization of Nucleobases in Broadband Terahertz Spectra from 0.5 to 10 THz with the Air-Biased-Coherent-Detection Technique

**DOI:** 10.3390/s19051148

**Published:** 2019-03-07

**Authors:** Miao Yu, Shihan Yan, Yong-qiang Sun, Wang Sheng, Fu Tang, Xiao-yu Peng, Yuan Hu

**Affiliations:** 1 Key Laboratory of Opto-electronic Measurement and Optical Information Transmission Technology, Ministry of Education, Changchun University of Science and Technology, Changchun 130022, China; yumiao@cigit.ac.cn (M.Y.); 13941974386@163.com (Y.-q.S.); 2 Chongqing Institute of Green and Intelligent Technology, Chinese Academy of Sciences, Terahertz Technology Research Center, Chongqing 400714, China; shengwang@cigit.ac.cn (W.S.); 20094235@cqu.edu.cn (F.T.); 3 Chongqing Institute of Green and Intelligent Technology, Chinese Academy of Sciences, Center of Applied Physics, Chongqing 400714, China; 4 Chongqing Engineering Research Center of High-Resolution and Three-Dimensional Dynamic Imaging Technology, Chongqing Institute of Green and Intelligent Technology, Chinese Academy of Sciences, Chongqing 400714, China

**Keywords:** THz-TDS, THz-ABCD system, fingerprint spectrum, nucleobase

## Abstract

Terahertz time-domain spectroscopy (THz-TDS) is an effective coherent detection technique for deeply understanding the structures and functions of biomolecules. However, generally not full information in the whole THz range can be obtained due to the limited detection bandwidth (usually less than 5 THz) of the traditional THz-TDS systems. In this paper, effective THz absorption spectra in 0.5–10 THz range of five typical nucleobases of DNA/RNA are characterized with a super broadband THz detection technique, called the air-biased- coherent-detection (THz-ABCD) technique. Few unexpected characteristic absorption peaks appeared in the low-frequency region and meanwhile a series of anticipated characteristic absorption peaks are found in the high-frequency region. The fingerprint spectra of these nucleobases are helpful for further analysis on the vibration and twisting behavior of hydrogen bonds, van der Waals and electrostatic forces etc. between and within DNA/RNA biomolecules.

## 1. Introduction

The spectra of biomolecules in the different electromagnetic bands contain abundant structural and functional information, which is meaningful and helpful for the qualitative and quantitative analysis of the structures and functions of target objects. For biological macromolecules, low frequency stretching and bending vibrations between and within molecules, phonon vibrations of lattices and stretching and torsion vibrations of hydrogen bonds pertain to the primary reasons of generating high-level structure and motion. Hydrogen bonds, van der Waals forces, and skeletal vibrations between or within nucleic acids, proteins, sugars and lipids that reflect the biological functions of biological molecules fall in the terahertz (THz) band range [1,2,3]. The determination of the biomolecules’ unique THz spectral fingerprints has been considered to be particularly important for material identification and theoretical research about biofunction [4,5,6].

With the rapid development of THz time-domain spectroscopy (THz-TDS), the optical parameters of bio-macromolecules below 4 THz have been obtained routinely [7,8]. However, it is still very difficult to get more complete and detailed fingerprint spectra in the full THz region using the current THz-TDS techniques due to the limited bandwidth of THz sources or/and the detection bandwidth [9,10], which limit the application of THz spectroscopy in biomedicine. The characteristic absorption peaks of biomolecules related to the bending vibrations of functional groups in biological macromolecules have been proved theoretically to be widely distributed in the whole THz band [11]. For example, characteristic peaks of histidine molecules at 1.19, 1.90, 2.57, 4.64, 6.79, 7.78 and 8.73 THz etc. are anticipated through theoretical simulation [12]. The THz sources based on femtosecond laser plasmas have made many breakthroughs in the power and bandwidth above 10 THz recently [13,14,15], but the detection techniques still provide unsatisfactory detection bandwidths, for example, the photoconductive antenna (PA) detection technique bandwidth is generally less than 3 THz due to its high frequency response limited by the carrier life time and the relaxation time [16]. With the electro-optical sampling (EOS) detection technique with zinc telluride (ZnTe) crystals or with gallium arsenide (GaAs) crystals is difficult to obtain the intact information of samples due to their intrinsic phonon absorption [17,18]. Recently, the technique of THz sources based on two-color-laser-induced air plasmas and a broadband coherent detection technique, called THz air-biased-coherent-detection (THz-ABCD) have been proposed [19,20,21,22] with breakthroughs of more power and much broader bandwidth of the THz source and detection range. This technique has drawn extensive attention, especially in biomedical studies [23,24] due to its significantly improved measurement range that covers the entire THz gap and even goes over 10 THz. This technique can provide more information of the basic crucial data of biostructures.

Adenine (A), cytosine(C), thymine (T), guanine (G) and uracil (U) are the basic components of DNA and RNA, which control protein synthesis based on the rule of base complementary pairing and ensure the accuracy of genetic biology [25]. The test results of these molecules’ spectra in the THz frequency band may become the basis of understanding the central dogma through the vibration and twisting information associated with the advanced structures. Up to now, spectral information of nucleobases above 5 THz was not available by using the traditional THz-TDS techniques. Besides, there are some notable differences in the results reported by different research groups [26,27,28,29]. The incompleteness and the uncertainty of spectral information for these base-pairs might affect the correct understanding of the DNA/RNA biomolecular structures and functions. In order to overcome these limitations and uncertainties, in our research, we use THz-ABCD system detect the five nucleobases (A, C, G, T and U) and calculate the optical parameters to obtain the absorption spectra in the broadband range from 0.5 to 10 THz. Compared with the previous studies, some new characteristic absorption peaks of the THz fingerprint spectra of the AGCTU nucleobases in the high frequency range from 5–10 THz are firstly found, and the peaks in the lower frequency range are repeated. Our results indicate that this technology is a powerful tool in the field of biochemistry, structural biology and structural bioinformatics, etc.

## 2. Experimental Procedure and Parameter Calculation

### 2.1. Experimental Procedure

**Figure 1:**
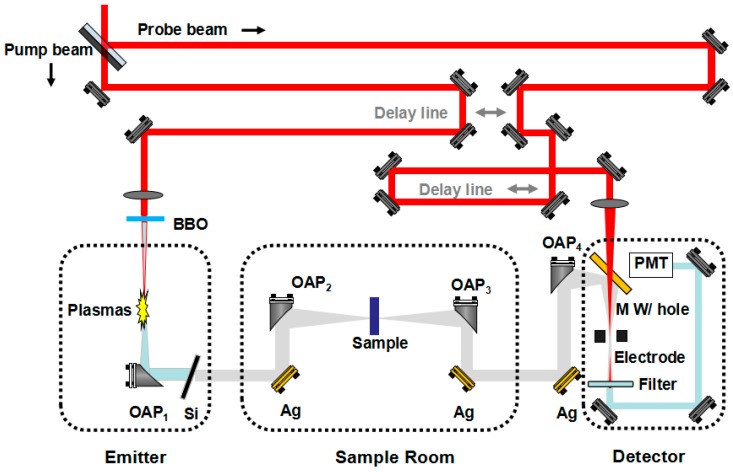
Experimental setup of the broadband THz-TDS based on the THz-ABCD technique.

The commercial Spitfire Ace titanium sapphire laser amplifier system (Spectra-Physics, Santa Clara, California, USA) delivers laser pulses with 10 mJ pulse energy, 50 fs pulse duration and 800 nm central wavelength at 1 kHz repetition rate. Only a 2.5 mJ pulse for generating and detecting THz wave is split from the 10 mJ pulse by a 75–25% broad bandwidth beam splitter, and then divided into the pump pulse and the probe pulse by a splitter. The 800 nm beam, 400 nm beam and THz beam are respectively marked as the red, blue and grey lines in the diagrammatic sketch (Figure 1). Then, the pump pulse is focused by a 100-mm-focal-length convex lens and generates a second harmonic (400 nm) after the pulse passes through a 100-µm-thick β-barium borate (BBO) crystal (Type-I). The fundamental laser pulse and its second harmonic are focused in the ambient air to generate plasmas, which induces transverse time-varying photocurrents in the plasmas, and then the broadband THz radiation is generated. The THz radiation is collected, collimated, and focused by four off-axis parabolic mirrors. A silicon wafer with high resistivity is placed in the THz transmission path to block the residual laser light and the super-continuous radiation from the plasmas. The probe laser beam is focused at the same point after passing through the mirror with a hole, where the THz field induces the laser second harmonic due to the four-wave mixing process [30]. A pair of linear electrodes are set across the focal spot where the THz beam and probe laser beam overlap, which provides an alternating biased field for the heterodyne detection of THz waves. The second harmonic pulses of the probe laser are measured with a photomultiplier (PMT) and then input to a lock-in amplifier. Samples are put in the focus of the second off-axis parabolic mirror (OAP_2_) for transmission measurement to obtain THz time domain waveforms.

### 2.2. Parameter Calculation

**Figure 2:**
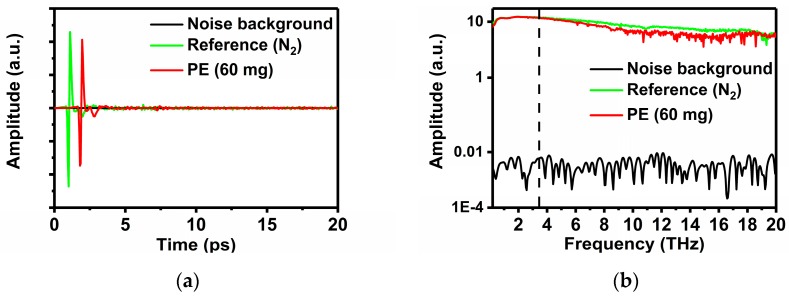
Waveforms of the noise background, reference and PE of ABCD system. (**a**) Time domain spectrum; (**b**) Fourier transmitted spectrum.

Spectroscopy measurements are performed using THz-ABCD as shown in Figure 1. The transmitted electrical field E(t) is sampled at about 3.3 fs in a time interval of 20 ps. A frequency spectrum of the signal is achieved by applying the Fast Fourier Transform (FFT) to the temporal pulse, achieving a resolution of about 50 GHz. In order to identify the origin of those fine peaks appeared in highly absorptive samples, background noise spectrum in dry conditions of ABCD system is shown in Figure 2. All measurements are performed in a purging box filled with continuously injected nitrogen (N_2_) in clean room with constant temperature at 295 ± 1 K and keeping around 10 min till the humidity level lower than 0.1% to eliminate the effect from the gaseous H_2_O absorption before each tests for reference and different samples. The nucleobases AGCTU and polyethylene (PE) are purchased from Sigma-Aldrich Company (Shanghai, China) and no further purification was conducted before use. The pure sample powder is squeezed into thin slices with a thickness between 0.3 mm to 0.7 mm and a diameter of 13 mm by using a manual tableting machine (hy-2A, Tianjin Tianguang Optical Instrument Co., Ltd., Tianjin, China). According to Mickan’s methods [31], the complex transmission coefficient can be expressed as:

(1) $$\tilde{T}=\frac{{\tilde{E}}_{sample}}{{\tilde{E}}_{reference}}=\rho (\omega )e^{-j\phi (\omega )}$$

The refractive index of the sample can be expressed as:

(2) $$n=\varphi \left(\omega \right)\frac{c_{0}}{\omega d}+1$$

The absorbance of the sample can be expressed as:

(3) $$\alpha =\frac{2}{d}\ln\frac{4n}{\rho \left(\omega \right){\left(1+n\right)}^{2}}$$

where *d* is the thickness of the sample, and *c*_0_ is the propagation speed of light in vacuum.

Theoretically, the THz absorption spectra of pure nucleobases can be calculated from Equations (1)–(3). In our experiments, the THz spectrum of 80 mg pure nucleobases and 50 mg pure nucleobases are obtained to observe these absorption peaks. In the case of 50 mg/80 mg pure sample, it is concluded that for some absorption peaks located in the strong absorption bands it is difficult to identify their exact peak positions. In order to obtain the whole reliable information of the characteristic absorption spectrum in almost entire THz range (0.5–10 THz) for each nucleobase, we reduce the mass of the sample to 20 mg for measurement to identify strong absorption peaks referring to previous reports [32]. In this case, the pure nucleobase powder and PE powder are mixed and pressed to a tablet evenly with a mass ratio of 3:1. It should be noted that the PE powder also absorbs and scatters THz radiation in a certain degree [24], especially above 3.5 THz as proved in our measurements shown in Figure 2.

Further, one more professional and suitable method based on the mean field theory (Landau–Lifshitz–Looyenga model) [33,34] was used to further eliminate the effect of PE absorption:

(4) $${\tilde{\varepsilon }}_{nucleobase}(\eta_{nucleobase})={\left({\tilde{\varepsilon }}_{mixture}^{1/3}-(1-\eta_{nucleobase})\cdot {\tilde{\varepsilon }}_{polyethylene}^{1/3}\right)}^{3}/\eta_{nucleobase}^{3}$$

Here, ${\tilde{\varepsilon }}_{nucleobase}$ represents the complex permittivity of the nucleobase and $\eta_{nucleobase}$ is concentration in volume.

$\eta_{nucleobase}$ can be easily expressed in terms of the weight concentrations $\gamma_{nucleobase}$ and $\gamma_{polyethylene}=1-\gamma_{nucleobase}$ and mass densities $\delta_{nucleobase}$ and $\delta_{polyethylene}$, respectively:

(5) $$\eta_{nucleobase}={\left(1+\frac{\delta_{nucleobase}}{\delta_{polyethylene}}\cdot \frac{1-\gamma_{nucleobase}}{\gamma_{nucleobase}}\right)}^{-1}$$

The relation between optical parameters and complex permittivity can be expressed in the terahertz band as [35,36,37,38]:

(6) $$k=c\alpha /2\omega \;\tilde{\varepsilon }=\varepsilon_{r}+i\varepsilon_{i},\;\varepsilon_{r}=n^{2}-k^{2},\;\varepsilon_{i}=2nk$$

The parameters of the base sample were extracted from the mixture according to Equations (4) and (5). According to [39,40], we can define an uncertainty for each couple of functions in and $\varepsilon_{r}$ and $\varepsilon_{i}$ relative to a fluctuation of 1 μm of the optimal sample thickness *d*. This reflects in an error of the order less than 0.5% for each curve of each nucleobase. In order to reduce the measure error, ten THz time-domain signals are averaged for one sample as ${\tilde{E}}_{sample}$ at Equation (1) to calculate the absorbance, and 10 tablet samples per each nucleobase molecule are tested in each group. And more detailed samples characteristics are provided in Table 1 for analyzing the relevance of the sample thickness to extract electrodynamic information of samples.

## 3. Experimental Results

**Figure 3:**
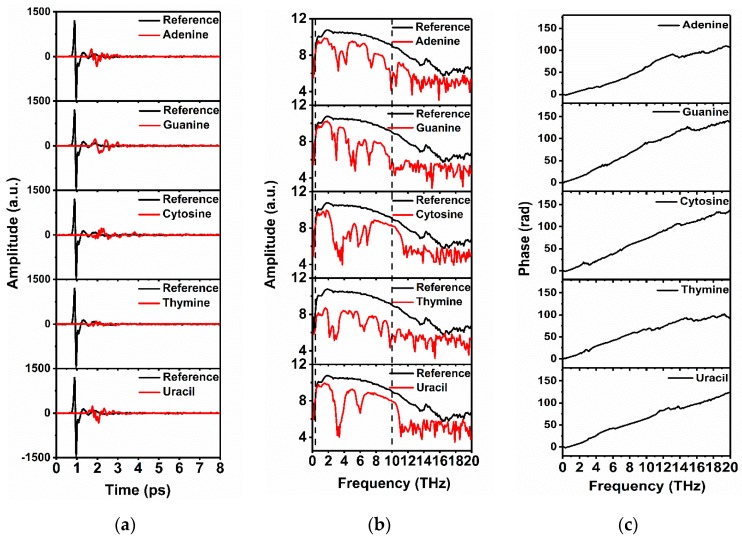
Waveforms of the nucleobases (50 mg) and the reference. (**a**) Time-domain spectra; (**b**) Fourier transform spectra; (**c**) The phase spectra.

Experimental measurement of the time-domain transmitted waveforms of the reference and the nucleobases with different mass were performed with our THz-ABCD system. Here we only present time (Figure 3a), frequency domain signals (Figure 3b) and phase (Figure 3c) of five pure nucleobases with 50 mg mass in the band (0.5–20) THz for understanding the preliminary measurements before calculating the absorption spectra.

From Figure 3a, it can be observed that the time-domain signals amplitude of nucleobases are attenuated substantially due to the absorption of THz radiation by each nucleobase compared with the reference signal. The phase delay can also be observed due to the relatively bigger refractive index of the nucleobases compared to N_2_. Figure 3b shows the corresponding Fourier transform spectra from the time-domain signals and the corresponding phase shown in Figure 3c. These frequency-domain spectral curves are continuous and smooth within a certain region, and all the transmission spectra of nucleobase samples are attenuated due to the resonance absorption. It is difficult to detect THz signal accurately below 0.5 THz due to its low amplitude within the f 0.1 to 0.5 THz range Meanwhile, the THz transmission spectral curve is continuous and smooth as seen from Figure 3b and displays a smooth increasing phase in Figure 3c in the 0.5–10 THz region, which indicates that a good signal-to-noise ratios (SNRs) in the range of 0.5–10 THz can be guaranteed in this study. In fact, the absorption spectra to some samples located over 10 THz are still credible due to the considerable SNRs, and the information over 10 THz is discarded just for the consistency of analysis in this work.

**Figure 4:**
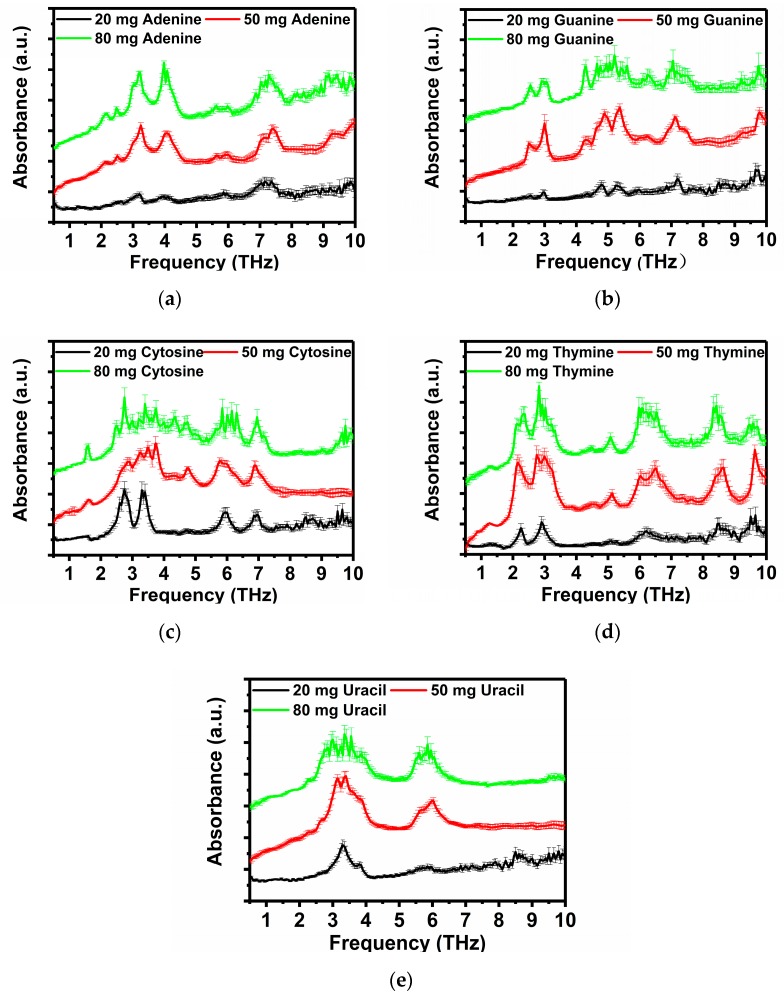
Absorption spectra of DNA/RNA nucleobases with three different mass. (**a**) Adenine; (**b**) Guanine; (**c**) Cytosine; (**d**) Thymine; (**e**) Uracil.

The absorption spectrum of each nucleobase is calculated according to Equations (1)–(6). The absorption spectrum of the nucleobases of DNA/RNA, A, G, C, T and U at masses of 80 mg, 50 mg and 20 mg for each nucleobase are shown in Figure 4. Nearly all existent absorption peaks with different strength could be identified clearly under various sample mass. Figure 4 shows the absorption spectra of five types of purine/pyrimidine samples in the mass of 20 mg, 50 mg and 80 mg mass. In the case of mixture samples of 20 mg nucleobase and 60 mg PE, exact positions of the absorption peaks located in the strong absorption bands can be identified. Subsequently, when the mass is increased to 50 mg or even 80 mg, those absorption peaks located in the weak absorption bands could become much clearer and the exact positions of more vibration absorption peaks can be observed.

For A (Figure 4a), no obvious absorption peak appears except those located at 3.10, 4.10 and 7.30 THz in the case with 20 mg A. When the mass is 50 mg/80 mg, the more positions of vibration absorption peaks can be observed at 1.70, 2.15, 2.50, 3.10 and 4.10 THz, which is consistent with previous literature reports. For G (Figure 4b), only four absorption peaks at 3.00, 4.80, 5.35 and 7.20 THz can be observed in the case of 20 mg mass, and these four absorption peaks become stronger and other absorption peaks at 2.50, 4.30, 6.30 and 9.80 THz can be identified much more clearly when the mass is 50 mg or 80 mg. Particularly, in the case of 80 mg G, there are many spikes in the band from about 4.50 to 5.50 THz, interfering with the identification of the positions of the absorption peaks at 4.30, 4.80 and 5.35 THz. For C (Figure 4c), four explicit absorption peaks at 2.75, 3.40, 5.95 and 6.95 THz can be identified when the mass is 20 mg and other absorption peaks at 1.55 and 4.75 THz are observed when the mass is increased to 50 mg. Moreover, a new peak at 4.35 THz is detected when the mass is further increased to 80 mg, but meanwhile, a lot of jittery spikes arise in the bands of 2.50–4.00 THz and 5.50–6.50 THz when the mass is 50 mg/80 mg. For T (Figure 4d), there are five absorption peaks located at 2.25, 2.95, 6.30, 8.50 and 9.60 THz in the case of 20 mg. Other absorption peaks at 1.30, 4.50 and 5.10 THz can be observed in the cases with higher masses. Among them, the characteristic absorption peaks observed in the band of 0.50–3.00 THz are consistent with previous literature reports. Figure 4e shows the THz absorption spectra of U, a special nucleobase for RNA. In the frequency interval of 2.50–4.00 THz and 5.50–6.50 THz, the vibration absorption of U is very strong. Therefore, characteristic absorption peaks located at 3.30 THz and 5.90 THz can be observed even in the case of a small mass sample (20 mg), but the absorption peaks at 2.30, 2.70 and 3.80 THz are extremely weak, and can only be observed by increasing the mass to 50 mg and 80 mg.

## 4. Discussion

All the absorption peaks of five nucleobases of DNA/RNA measured in this work and other reports previously are summarized in Table 2 for further comparison and analysis [26,27,28,29].

THz absorption spectra reflect the transition of electron energy levels from the ground state to the excited state by the THz radiation absorbed by molecules or atoms. It is accompanied by the transition of vibrational and rotational modes. Within the 0.5–10 THz range, the collective vibration of molecules, and the hydrogen bond and weak interaction between molecules contribute greatly to the vibration modes of A, G, C, T, and U [11]. There is a great difference in intensity of the resonance absorption between different molecule systems under the different frequencies. The weak absorption peaks at some frequencies become clear gradually with the increase of mass, but at some frequencies, the strong absorption leads to a lot of jittery spikes which hinder distinguishing the exact positions of absorption peaks, so then referring [26,27,28,29], we mixed in PE to dilute the mass of tested objects for measurement and analysis. Therefore, this technique has a great significance to detect different masses of samples to obtain complete characteristic absorption peaks in THz time-domain spectrum analysis.

The results of characteristic peaks of nucleobases using THz-ABCD are not only highly consistent with previous reports, but also over their limited detection range. Some minor differences of results may be caused by the inhomogeneity of powder, sample production method differences or measurement errors [41,42]. Apart from the fact the absorption peaks in the low-frequency bands are repeated, several new molecular characteristic absorption peaks are found experimentally in the higher frequency bands with the THz-ABCD technique, which could correspond to the theoretical simulations. This work mainly supplements the absence of 5.0 - 10 THz frequency band based on the previous reports and the result demonstrates the powerful detection capabilities of the full THz spectrum by THz-ABCD.

Nevertheless, there are still a few special peaks not always appearing except in the results of individual groups. Presumably, these absorption peaks, at 3.44 THz and 3.89 THz of A, at 5.30 THz of C and at 1.69 THz of U, claimed in [27] are not observed in our work or others and these peaks also cannot be distinguished clearly in Figure 2a in [27]. These “unique case” peaks may be noise signals because of the large mass of the sample pellets in [27] among these mentioned works [26,27,28,29], or they could have originated from the interaction between purine and the water molecules during recrystallization [43]. The characteristic absorption peaks at 2.07 and 2.82 THz of T claimed in [28] are not repeated in our study or others. These peaks are extremely weak, laying on the shoulders of nearby peaks, respectively, and may be interfered and covered by the vibration caused by the nearby strong peak bands. Besides, contiguous peaks located at close frequencies with distinct intensity are often present as one wider absorption envelope when the spectral accuracy is insufficient, which may be the reason that our work or other results failed to detect a 2.53 THz absorption peak of C [29]. The samples in [28] and [29] contained a smaller mass of nucleobases because they are mixed with a lot of PE in a thinner thickness compared to our setting, which would help enhance these weak peaks by avoiding too strong absorptions of the close peaks. Moreover, the absorption peak at 3.43 THz of T in [29] is also not repeated by our work or others. This characteristic frequency is very close to the detection limit of spectrum range based on the electro-optical sampling (EOS) detection technique, and may actually be the beginning of the noise signal, but beyond that, there is an unexpected peak at 4.5 THz for T discovered firstly below the 5.0 THz region. As shown in Figure 4d, this absorption of T at 4.5 THz is weak, and the peak becomes clear as the mass increases, which could easily be ignored as noise when the mass of T is not enough or the detector is not sensitive enough. In any case, further authentication analysis is required in the future with higher resolution THz-TDS in order to truly distinguish the real characteristic absorption signals.

Our results indicate that THz-ABCD technique is superior to the conventional THz-TDS in terms of bandwidth, and it can provide more information about molecular vibrations and twists, which is helpful for identification of substances and analysis of biomolecules’ structures. These absorption peaks are related to different nucleobase molecular vibration modes. This information also contributes to the improvement of theoretical simulations.

## 5. Conclusions

In conclusion, the THz absorption spectra of nucleobases made up DNA/RNA are measured by a THz ABCD system with an effective frequency band from 0.5 to 10 THz. In contrast to previous reports, we found several new characteristic absorption peaks not only in the high THz frequency band, but also in the low frequency band. These results indicate that the THz-ABCD technique has a unique advantage to obtain more detailed detection information over a wider detection range in the research field of spectral analysis of biological macromolecules compared to other THz-TDS techniques. As the bases of the nucleic acids control the growth, development, differentiation and death processes or organisms, the improvement of their THz spectral database will be helpful for the further analysis and investigation on their resonance, rotation and other behaviors.

## Figures and Tables

**Table 1:** Sample characteristics.

	Sample Name	Sample Ingredient	Mass Ratio (%)	Thickness (mm)
80 mg	A	Pure adenine	100	0.594
	G	Pure guanine		0.588
	C	Pure cytosine		0.600
	T	Pure thymine		0.589
	U	Pure uracil		0.597
50 mg	A	Pure adenine	100	0.320
	G	Pure guanine		0.311
	C	Pure cytosine		0.307
	T	Pure thymine		0.340
	U	Pure uracil		0.319
20 mg	A	Mixture of 20 mg adenine and 60 mg PE	25	0.629
	G	Mixture of 20 mg guanine and 60 mg PE		0.634
	C	Mixture of 20 mg cytosine and 60 mg PE		0.626
	T	Mixture of 20 mg thymine and 60 mg PE		0.639
	U	Mixture of 20 mg uracil and 60 mg PE		0.633
	PE	Pure PE 60 mg		0.508

**Table 2:** The positions of absorption peaks of five nucleobases (unit: THz).

**A**	THz-ABCD	1.70	2.15	2.50	3.10			4.10	5.60	6.00	7.30		
	TDS [26]	1.7	2.2		3.1								
	TDS [27]	1.67	2.11	2.54	3.05	3.44	3.89	4.18					
	TDS [28]	1.75	2.09	2.5									
**G**	THz-ABCD	2.50	3.00	4.30	4.80	5.35	6.30	7.20	9.80				
	TDS [26]	2.5	2.9										
	TDS [27]	2.57	3.00	4.31	4.84	5.44							
	TDS [28]	2.52	2.99										
**C**	THz-ABCD	1.55		2.75	3.40	4.35	4.75		5.95	6.95			
	TDS [26]	1.6		2.7	3.3								
	TDS [27]	1.60		2.85	3.39	4.32		5.30					
	TDS [28]	1.59		2.73									
	TDS [29]	1.55	2.53	2.72	3.25								
**T**	THz-ABCD	1.30		2.25			2.95		4.50	5.10	6.30	8.50	9.60
	TDS [26]			2.3			2.9						
	TDS [27]	1.36		2.29			3.00			5.10			
	TDS [28]		2.07	2.29		2.82	2.94						
	TDS [29]	1.30		2.25			2.86	3.43					
**U**	THz-ABCD		2.30	2.70	3.30	3.80	5.90						
	TDS [27]	1.69	2.31	2.68	3.44	3.84							
